# Two Lineages of Papillomaviruses Identified from Caracals (*Caracal caracal*) in South Africa

**DOI:** 10.3390/v16050701

**Published:** 2024-04-29

**Authors:** Simona Kraberger, Laurel E. K. Serieys, Gabriella R. M. Leighton, Matthew D. De Koch, John S. Munday, Jacqueline M. Bishop, Arvind Varsani

**Affiliations:** 1 The Biodesign Center for Fundamental and Applied Microbiomics, Center for Evolution and Medicine and School of Life Sciences, Arizona State University, Tempe, AZ 85287, USA; mdekoch@asu.edu; 2 Panthera, 8 W 40th St, 18th Floor, New York, NY 10018, USA; lserieys@panthera.org; 3 Institute for Communities and Wildlife in Africa (iCWild), Department of Biological Sciences, University of Cape Town, Cape Town 7701, South Africa; lghgab001@myuct.ac.za (G.R.M.L.); jacqueline.bishop@uct.ac.za (J.M.B.); 4 School of Veterinary Science, Massey University, Tennant Drive, Palmerston North 4442, New Zealand; j.munday@massey.ac.nz; 5 Structural Biology Research Unit, Department of Integrative Biomedical Sciences, University of Cape Town, Cape Town 7925, South Africa

**Keywords:** *Caracal caracal*, *Papillomaviridae*, *Lambdapapillomavirus*, *Treisetapapillomavirus*

## Abstract

Papillomaviruses (PV) infect epithelial cells and can cause hyperplastic or neoplastic lesions. In felids, most described PVs are from domestic cats (*Felis catus*; n = 7 types), with one type identified in each of the five wild felid species studied to date (*Panthera uncia*, *Puma concolor*, *Leopardus wiedii*, *Panthera leo persica* and *Lynx rufus*). PVs from domestic cats are highly diverse and are currently classified into three genera (*Lambdapapillomavirus*, *Dyothetapapillomavirus*, and *Taupapillomavirus*), whereas those from wild felids, although diverse, are all classified into the *Lambdapapillomavirus* genus. In this study, we used a metagenomic approach to identify ten novel PV genomes from rectal swabs of five deceased caracals (*Caracal caracal*) living in the greater Cape Town area, South Africa. These are the first PVs to be described from caracals, and represent six new PV types, i.e., Caracal caracal papillomavirus (CcarPV) 1–6. These CcarPV fall into two phylogenetically distinct genera: *Lambdapapillomavirus*, and *Treisetapapillomavirus*. Two or more PV types were identified in a single individual for three of the five caracals, and four caracals shared at least one of the same PV types with another caracal. This study broadens our understanding of wild felid PVs and provides evidence that there may be several wild felid PV lineages.

## 1. Introduction

Papillomaviruses (PVs; family *Papillomaviridae*) are circular double-stranded (dsDNA) viruses that infect mammals, birds, and reptiles [1,2,3,4]. Highly diverse and generally species-specific, PVs are epithelial-cell-trophic. A single host can be infected with several PV types, including types that are classified within different genera [5,6]. PV genomes are composed of five to six early genes and two late genes. The L1 gene which encodes the major capsid protein typically shows higher levels of conservation among PVs and is used for taxonomic classification with those sharing >60% L1 nucleotide similarity belonging to the same genus, >70% for species, and >90% for type [2]. Currently, most of the known PV types are those infecting humans, with a significant knowledge gap for non-human PVs and their broader evolution [2,4].

In felids, several unique papillomaviruses have been identified which can cause cutaneous or oral lesions [7]. The most studied feline species is the domestic cat (*Felis catus*). In domestic cats, PVs rarely cause the hyperplastic papillomas (warts) which are common in other felid species [7]. Instead, many PV types are detected in association with preneoplastic viral plaques or invasive neoplasms of the skin [7,8]. Seven PV types from domestic cats have been classified to date: Felis catus papillomavirus (FcaPV) 1–7 [9,10,11,12,13,14,15]. FcaPV7 (OL310516; [16]), although identified from a skin swab from a human, is thought to be of feline origin due to the person being a cat owner and the genome showing similarities to FcaPV2. In addition, an unclassified FcaPV genome (OQ836188; [17]) was recovered from an infection associated with skin cancer in a domestic cat that will likely be FcaPV8, and another, Bos taurus papillomavirus (BPV) 14 [18], was found to cause feline sarcoids after cross-species infection from its bovine host [19] (Table 1). The FcaPVs belong to three different genera, *Lambdapapillomavirus* (FcaPV1), *Dyothetapapillomavirus* (FcaPV2), and *Taupapillomavirus* (FcaPV3, -4, -5, -6), and FcaPV7 is currently unclassified but sits with FcaPV2 and is, therefore, likely a *Dyothetapapillomavirus*. In wild felid species, complete PV genomes have previously been documented in a bobcat (*Lynx rufus*)—Lynx rufus papillomavirus 1 (LrPV1), Asiatic lion (*Panthera leo persica*)—Panthera leo persica papillomavirus 1 (PlpPV1), snow leopard (*Uncia uncia* or *Panthera uncia*)—Uncia uncia papillomavirus 1 (UuPV1), mountain lion (*Puma concolor*)—Puma concolor papillomavirus 1 (PcPV1) [20,21], and margay (*Leopardus wiedii*)—Leopardus wiedii papillomavirus 1 (LwiePV1) [22]. These feline PV types all belong to the *Lambdapapillomavirus* genus, which has other PVs from the Carnivora species. Several partial felid PV sequences representing novel papillomaviruses have also been identified (Table 1) from domestic cats, cheetahs (*Acinonyx jubatus*) [23], the African lion (*Panthera leo*) [23], and snow leopards [24,25].

The monophyletic nature of the PVs identified from wild felid species contrasts with those from domestic cats which are polyphyletic [9,10,12,13,31]. This could be a result of several factors, such as the sampling bias of domestic cats, and/or to areas where obvious lesions are observed, as well as the geographic or region endemicity of the different wild felid species versus the broad global distribution and free ranging capability of domestic cats, exposing them to a higher cross-species transmission potential. The *Lambdapapillomavirus* feline lineage appears to be slow-evolving, at a rate of 1.95 × 10^−8^ nucleotide substitutions per site per year, and shows evidence of a long coevolutionary history with their feline host species [20]. Further, these viruses appear to have a unique second non-coding region between the early and late protein region [20].

Here, we identify ten complete PV genomes from five caracals (*Caracal caracal*) inhabiting the greater Cape Town area, Western Cape, South Africa. These are the first documented PVs from caracals and they belong to two genera, *Lambdapapillomavirus* and *Treisetapapillomavirus*, thus expanding our current knowledge on PVs in wild felids.

## 2. Materials and Methods

### 2.1. Study Site and Sample Collection

As part of a long-term study undertaken by the Urban Caracal Project (www.urbancaracal.org; accessed 12 February 2024) to monitor the health and well-being of caracals in the greater Cape Town area, rectal swab samples were collected during post-mortem of deceased animals (n = 26) between 2021 and 2023 using PurFlock ultra 6″ sterile flock swabs (Puritan Medical Products, Guilford, ME, USA). Cause of death for these caracals was determined to be motor vehicle impact when crossing urban roads, disease and/or poisoning, or poaching. The swabs were stored at −20 °C in Puritan UniTranz-RT media (Puritan Medical Products, Guilford, ME, USA) for downstream nucleic acid extraction.

### 2.2. Sample Processing and Papillomavirus Genome Identification

High Pure Viral Nucleic Acid Kit (Roche Diagnostics, Indianapolis, IN, USA) was used to isolate viral nucleic acid from 200 µL of the UTM buffer in which the swabs were stored. Then, 1 µL of the viral nucleic acid was enriched for circular molecules by rolling circle amplification (RCA) using the Illustra TempliPhi Kit (Cytiva Lifesciences, Marlborough, MA, USA). An aliquot of viral nucleic acid was combined with the RCA product and high-throughput sequencing (HTS) libraries were generated using an Illumina DNA prep (M) tagmentation kit (Illumina, San Diego, CA, USA). Libraries were sequenced on Illumina NovaSeq X plus sequencer at Psomagen Inc. (Rockville, MD, USA). The raw paired-end reads (2 × 150 bp) were trimmed using Trimmomatic −0.39 [40] and de novo assembled with MEGAHIT v1.2.9 [41]. The de novo assembled contigs of >1000 nts were screened against a viral RefSeq protein sequence database (release 220) using DIAMOND BLASTx [42]. We also screened the contigs for host mitochondrial genomes using Diamond BLASTx [42] with a mitochondrial RefSeq database (release 220). Contigs were determined as circular based on terminal redundancy. Read mapping to confirm adequate depth and coverage of full genomes was performed using BBmap [43].

The complete papillomavirus and mitochondrial genome sequences are deposited in GenBank with accession # OR915584-OR915593 and PP566117-PP566121, respectively. The SRA data are deposited under BioProject #PRJNA1045660, BioSample # SAMN38451862-SAMN38451866 and SRA # SRR26982246-SRR26982250 for the PV sequences. For the caracal mitochondrial genomes under BioProject # PRJNA1033669-PRJNA1033673, BioSample # SAMN38451862-SAMN38451866, and SRA # SRR28492289-SRR28492293.

### 2.3. Genome Characterization, Pairwise Comparison, and Phylogenetic Analyses

The PV genomes were annotated with Cenote-Taker2 [44], and then manually checked with annotation of PVs from PAVE [4]. The mitochondrial genomes of the hosts were annotated with the MITOS server [45,46], and then manually checked.

Pairwise similarity identities were determined for the full genome, and the E1, E2, E3, E6, E7, L1, and L2 genes and protein sequences of the PVs from this study and of those most closely related using SDT v1.2 [47].

Dataset of L1, E1, and E2 protein sequences of all PV types referenced at PAVE [4], as well as those from this study, were assembled. We opted to use the protein sequences of L1, E1, and E2 as these are the most conserved amongst the papillomaviruses and can be more credibly aligned than the corresponding nucleotide sequences. These were aligned using MAFFT [48] and trimmed using TrimAL [49] with the 0.2 gap option and concatenated (L1 + E1 + E2). The best-fit amino acid substitution models LG + I+G for the E1, LG + I + G + F for the E2, and LG + I + G + F for the L1 were determined using ProtTest3 [50]. A partitioned phylogenetic tree was inferred using IQ-TREE2 [51] with aLRT branch support and rooted with the L1 + E1 + E2 of avian papillomaviruses. The phylogenetic tree was visualized in iTOLv6 [52]. Mitochondrial genomes from caracals together with those available from other members of the *Caracal* genus available in GenBank were aligned using MAFFT [48] and a neighbor-joining tree was constructed using FastTree [53], implemented in Geneious Prime 2024.0.4.

The motif discovery and comparison tools MEME [54] and Tomtom [55] were used to identify conserved motifs in the non-coding regions of the genomes of feline-infecting *Lambdapapillomaviruses*.

## 3. Results and Discussion

### 3.1. Identification of Papillomavirus Genomes in Wild Caracal

**Figure 1:**
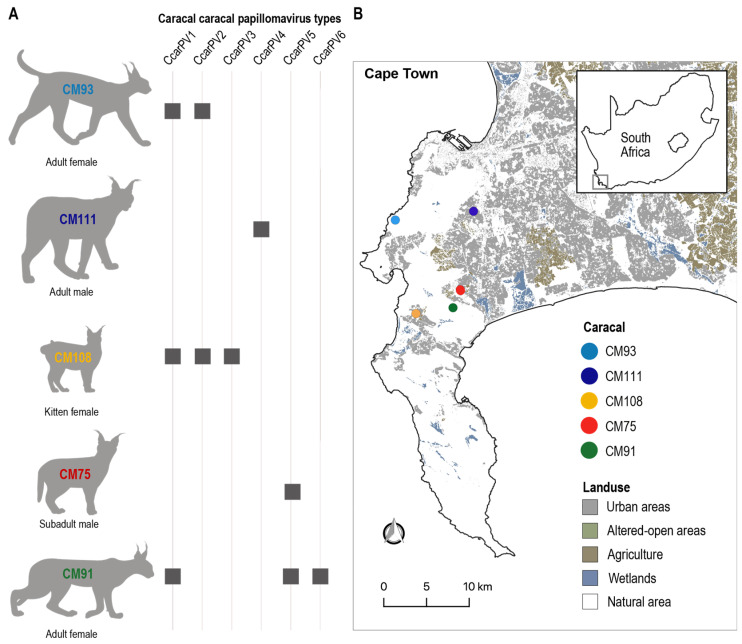
(**A**) Summary of caracal host and papillomavirus type. (**B**) Land-use map showing sampling locations of caracals that were found to be positive for papillomavirus.

As part of an ongoing effort to monitor caracal health and survival in the greater Cape Town area (Western Cape, South Africa), rectal swabs were collected from deceased caracals as a result of being hit by motor vehicles, and/or suspected disease or pesticide poisoning between the period 2021–2023 (Table 2). Ten novel PV genomes were determined from the rectal swabs of five caracals (Figure 1, Table 2). No obvious lesions or papillomas were observed during post-mortem sample collection.

**Figure 2:**
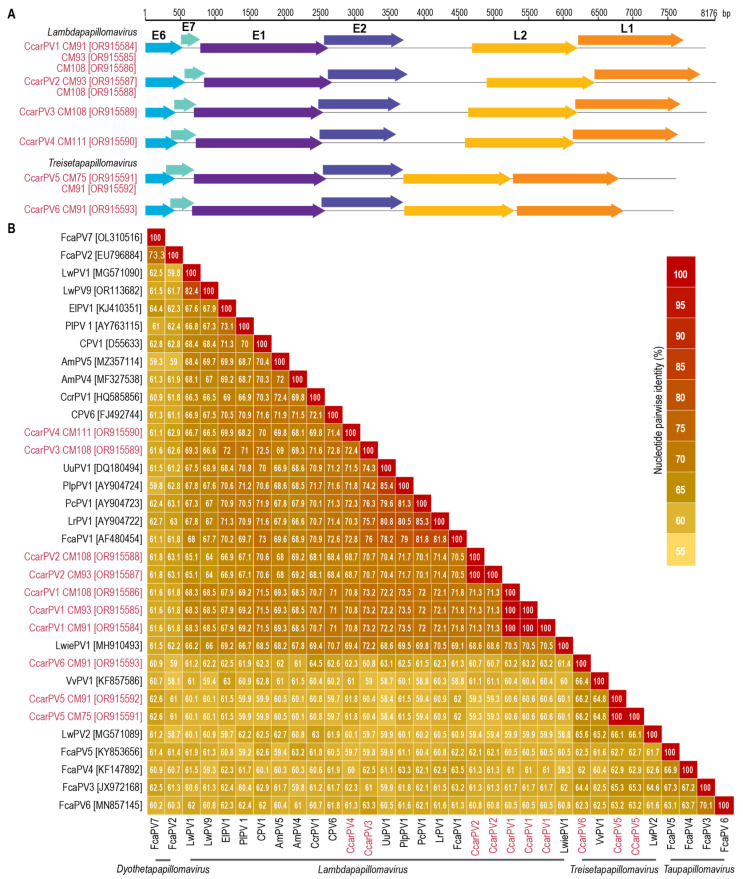
(**A**) Genome organization of CcarPV genomes described in this study. (**B**) L1 gene nucleotide pairwise comparisons of the CcarPVs with those of PVs that are members of Dyothetapapillomavirus, Lambdapapillomavirus, Taupapillomavirus (felid PVs only), and Treisetapapillomaviruss genera. PVs from caracals are shown in red. Abbreviations are as follows: Caracal caracal papillomavirus (CcarPV), Felis catus papillomavirus (FCaPV), Canis familiaris papillomavirus (CPV) Leptonychotes weddellii papillomavirus (LwPV), Crocuta crocuta papillomavirus (CcrPV), Vulpes vulpes papillomavirus (VvPV), Lynx rufus papillomavirus (LrPV), Puma concolor papillomavirus (PcPV), Panthera leo persica papillomavirus (PlpPV), Panthera uncia papillomavirus (UuPV), Leopardus wiedii papillomavirus (LwiePV), Ailuropoda melanoleuca papillomavirus (AmPV), Procyon lotor papillomavirus (PlPV), and Enhydra lutris papillomavirus (ElPV).

Ten PV genomes were de novo assembled from high-throughput sequencing data with an average read depth ranging from 21- to 108,899-fold. All ten PV genomes have identifiable E1, E2, E6, E7, L1, and L2 genes and range in genome size from 7566 to 8176 bp (Figure 2A). Based on the papillomavirus ICTV species demarcation determination of <70% pairwise identity for the L1 gene sequences and <10% pairwise identity for PV types, these ten PV represent six novel types; Caracal caracal papillomavirus (CcarPV) 1–6, (Figure 2, Table 2). CcarPV1, -2, -3, and -4 share >70% L1 gene sequence identity with other felid PVs, i.e., LwiePV1 [22], LrPV1, PlpPV1, UuPV1 and PcPV1 [20,21], and FcaPV1 [11], and, therefore, these all belong to the same species. CcarPV5 and -6 share <70% L1 gene nucleotide sequence identity with other PVs and each other, and, therefore, will represent two new species. All CcarPV isolates belonging to the same type share 100% L1 gene nucleotide sequence pairwise identity.

Caracals CM93 and CM91, both adult females, were found to have multiple PVs, with CM93 harboring two PVs, CcarPV1 (OR915585) and CcarPV2 (OR915587), and CM91 three PVs, CcarPV1 (OR915584), CcarPV5 (OR915592), and CcarPV6 (OR915593) (Figure 1 and Figure 2). Caracal CM108, a female kitten, harbored three PVs: CcarPV1 (OR915586), CcarPV2 (OR915588), and CcarPV3 (OR915589). Lastly, Caracal CM75 and CM111, both males, a subadult and an adult, both harbored one PV, CcarPV5 (OR915591) and CCarPV4 (OR915590), respectively. CM93, CM108, and CM91 were all infected with CcarPV1 and one or two other PV types, showing a high rate of PV coinfection which is commonly seen in mammals [6,56]. Further, CcarPV1 and -2 were present in both CM93 and CM108. Taking into consideration the fact that these three individuals share at least one PV type and were found deceased within a 10 km radius from each other, this may indicate these cats were related, and/or interacted with other caracal(s) not sampled infected with these PV types.

### 3.2. Caracal Mitochondrial Genomes

**Figure 3:**
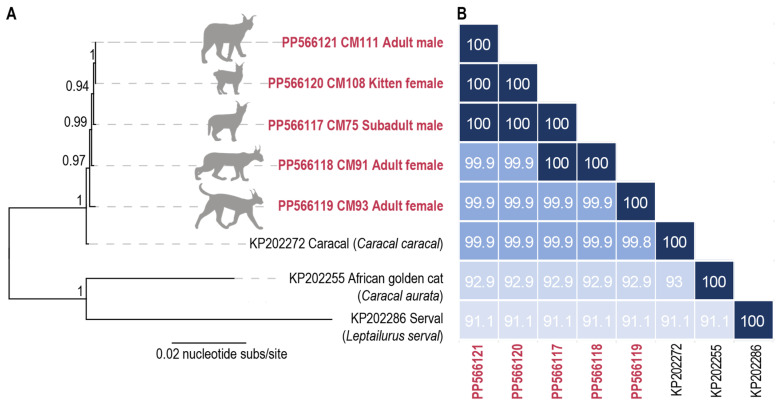
(**A**) Phylogeny of mitochondrial genomes from caracals in this study together with those in the caracal lineage available in GenBank. (**B**) Pairwise similarity of the mitochondrial genomes.

An advantage to using rolling circle amplification for the enrichment of circular DNA molecules is that this enables the simultaneous identification of host mitochondrial genomes that are also circular. From the PV-positive caracal samples, we were able to determine the full mitochondrial genomes. Phylogenetically (based on the mitochondrial sequences), all five caracals are closely related and sit within the Caracal clade, forming a sister lineage to the caracal mitochondrial genome (KP202272) available in GenBank (Figure 3). The mitochondrial genomes of caracals CM75, CM91, CM93, CM108, and CM111 share 99.9–100% pairwise nucleotide identity with each other and 99.8–99.9% pairwise nucleotide identity with a caracal mitochondrial genome (KP202272) [57] (Figure 3). This high level of similarity is not surprising given the recent study which showed that the Caracal population in Cape Town have elevated levels of inbreeding [58]. A comparison with mitochondrial genomes of two other members of the caracal lineage, an African golden cat (*Caracal aurata*) (KP202255) and a serval (*Leptailurus serval*) (KP202286) [57], showed they share 91.1–93% pairwise nucleotide identity.

### 3.3. Sequence Comparison of Caracal PVs

For the six CcarPV types, their genomes share 59.4–71.1% pairwise identity (Supplementary Data S1), showing a significant diversity amongst these genomes. For CcarPV1, CcarPV2, and CcarPV5, however, multiple isolates were identified from more than one individual, and, within each type, the isolate sequences are identical. PVs can be very slow-evolving, and, therefore, it is not uncommon to find identical sequences in samples from different individuals, even for PVs sampled decades apart [59,60]. A full-genome pairwise comparison of the CcarPVs with the PVs most closely related reveals that they share 58.8–72.6% pairwise identity, with CcarPV3 and PcPV1 (AY904723) from a puma [20] sharing the highest pairwise identity of 72.6%. A pairwise comparison of the protein sequences of E1, E2, E3, E6, E7, L1, and L2 for CcarPVs with those of PVs most closely related show that the L1 and E1 proteins share the highest pairwise identities ranging from 47.2–87.1% and 45.1–76.8%, respectively. Overall, the E6 protein has the lowest pairwise identity (23.1–59.1%) for the CcarPVs and those of the most closely related PVs.

### 3.4. Caracal PV L1 + E1 + E2 Phylogeny

**Figure 4:**
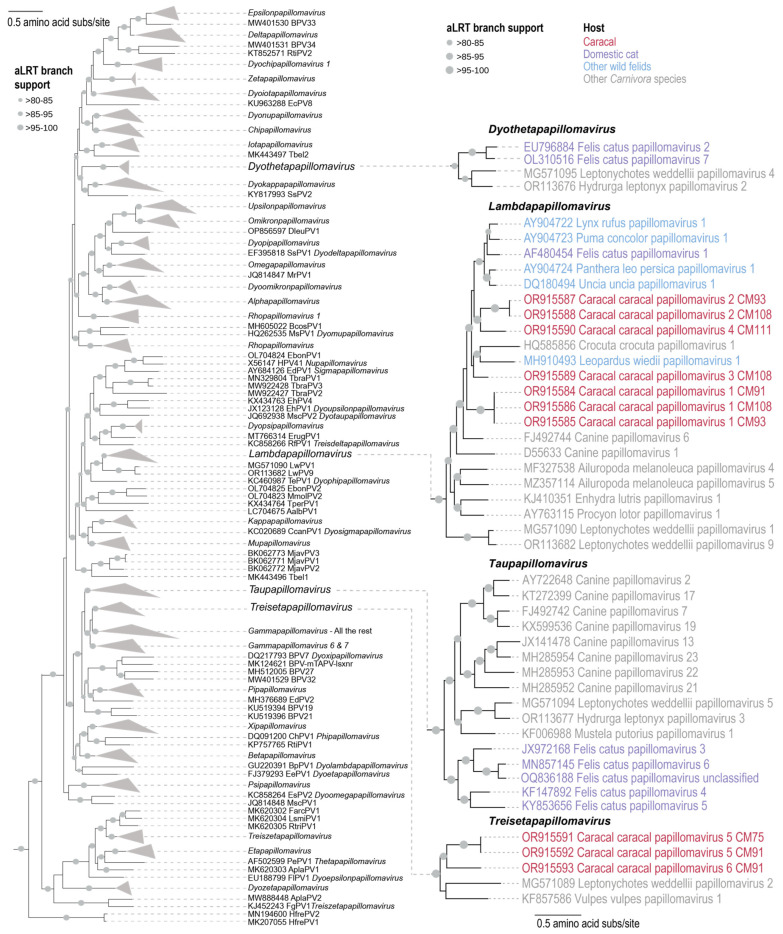
Maximum-likelihood phylogenetic tree of concatenated L1 + E1 + E2 protein sequences of the CcarPVs and representative PVs. PVs from caracals are shown in red font, those from domestic cats in purple, other wild felids in blue, and other Carnivora species in grey.

A maximum-likelihood phylogenetic tree was constructed from the concatenated L1 + E1 + E2 protein sequences of the caracal PVs and those of representative PV sequences from GenBank. This analysis showed these six CcarPV types are part of two genera, *Treisetapapillomavirus* and *Lambdapapillomavirus* (Figure 4). *Treisetapapillomavirus* currently comprises two PVs; one identified from a Weddel seal (*Leptonychotes weddellii*) [5] and one from a red fox (*Vulpes vulpes*) [61]. *Lambdapapillomavirus* comprises PVs from felid species (wild and domestic) [20,22], Weddel seal [5], giant panda (*Ailuropoda melanoleuca*) [32], sea otter (*Enhydra lutris*) [62], raccoon (*Procyon lotor*) [63], spotted hyena (*Crocuta crocuta*) [64], and domestic dog (*Canis familiaris*) [65,66]. Previously identified PVs from wild felids, puma and bobcat [20,22], all cluster with members of the *Lambdapapillomavirus* genus, whereas those from domestic cats are distributed across three genera, i.e., *Lambdapapillomavirus*, *Taupapillomavirus*, and *Dyothetapapillomavirus*. The CcarPVs from this study that are part of the *Lambdapapillomavirus* genus are CcarPV1, -2, -3, and -4, all grouped in a felid-PV-dominant subclade with one non-felid PV, CcrPV1 (HQ585856) from a spotted hyena [64]. CcarPV1 is basal in this clade, whereas CcarPV3 is most closely related to LwiePV1 (MH910493) [22] from a margay and CcrPV1 (HQ585856) from a spotted hyena [64]. CcarPV2 and -4 cluster in a clade that is basal to the other felid PVs in the genus *Lambdapapillomavirus* [11,20,22]. It should be noted that some of these subclades do not have strong branch support, and, therefore, as more PVs are identified and added to this group, it will likely help resolve these phylogenetic relationships more robustly. CcarPV5 and -6 group with the PVs in the genus *Treisetapapillomavirus* as a sister clade to Leptonychotes weddellii papillomavirus 2 (MG571089) [5] from a Weddell seal and Vulpes vulpes papillomavirus 1 (KF857586) [61] from a red fox. The polyphylogenetic distribution of the CcarPVs is similar to that noted for the domestic cat PVs, and, therefore, with the increased sampling of wild felids, a similar pattern may emerge. This is significant as it indicates a more complex evolutionary history than what was previously thought.

### 3.5. Large Non-Coding Region in the Genomes of Lambdapapillomaviruses

**Figure 5:**
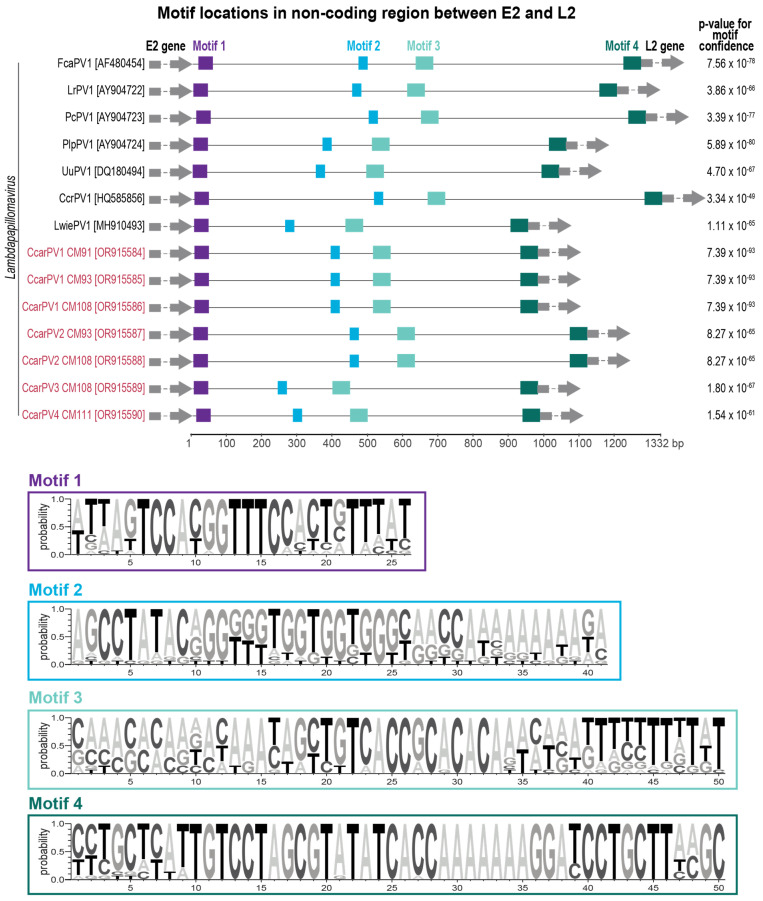
Conserved motifs identified in the non-coding region between the E2 and the L2 of the felid PVs and CcrPV1 from spotted hyena in the *Lambdapapillomavirus* genus. *p*-value indicates motif confidence.

Treisetapapillomavirus genomes are up to 1215 bp smaller (7392–7598 bp; [5,61] than those of lambdapapillomaviruses (7944–8607 bp) [11,20,64]. This difference in genome size appears to be due, at least in part, to a stretch of a non-coding region between the E2 and the L2 coding open reading frames (ORFs) in lambdapapillomaviruses, with the exception of Leptonychotes weddellii papillomavirus 1 (MG571090) from a Weddell seal [5]. This non-coding region has previously been noted and discussed in Rector et al. (2007). They noted there are several conserved regions that are likely to be of regulatory or other functional importance. To investigate this further, we used the motif discovery tool MEME [54] to scan this region, revealing four conserved motifs that were present in all the felid PVs and the CcrPV1 from spotted hyena (HQ585856) [64] in the *Lambdapapillomavirus* genus (Figure 5). A comparison of these regions with motif analyses tools such as Tomtom [55] indicates that these are possibly single-stranded DNA-binding motifs sharing the highest similarities to those associated with transcription factors in humans [67]. Although these findings support that this region has conserved motifs that may be involved in the DNA binding of transcription factors, in vitro molecular studies are needed to investigate this further.

### 3.6. E6 and E7 Protein Motifs

**Figure 6:**
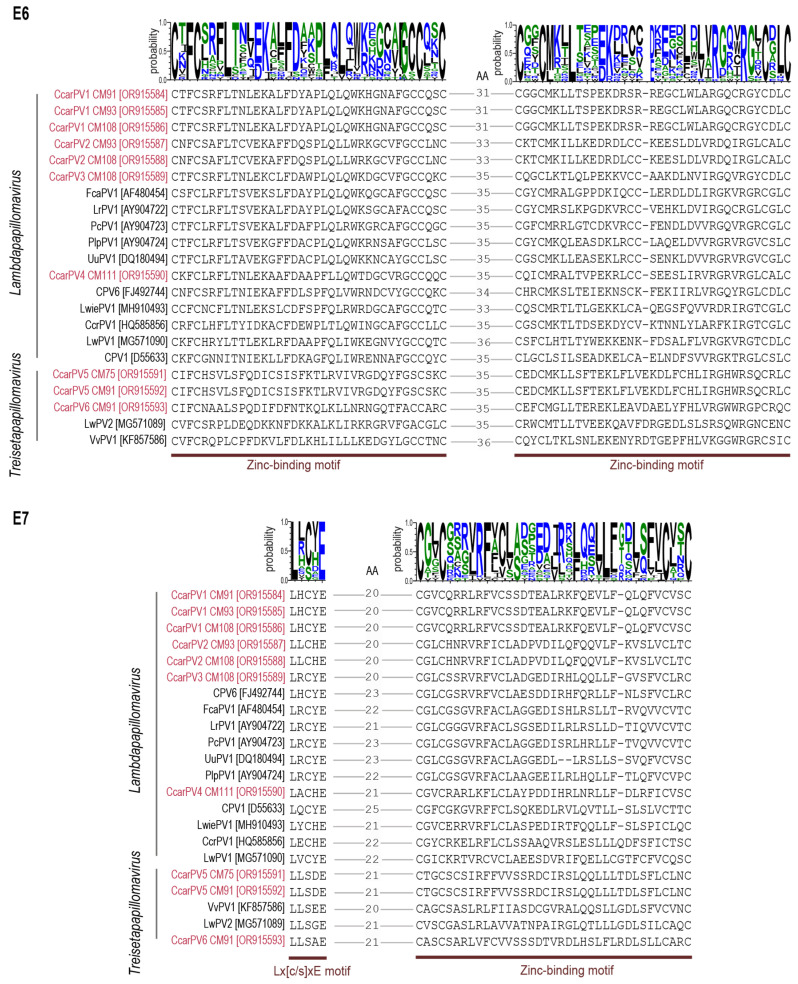
Zinc-binding motifs identified in the E6 proteins of treisetapapillomaviruses and lambdapapillomaviruses, and the pRB-binding motif (Lx[C/S]xE) and zinc-binding motif in E7 protein of the CcarPVs (red font) and those of the most closely related PVs. Hydrophobicity of amino acids is shown as follows: blue (hydrophilic), black (hydrophobic), and green (neutral).

The E6 and E7 are two early proteins that are encoded in most mammalian PVs. These oncoproteins have largely been studied in human PVs and play an important role in regulating the cell cycle in order to sustain cellular replication activity and viral proliferation [68]. Further, it is the ability of E6 and E7 proteins to bind tumor suppressors p53 and pRB, respectively, which is thought to drive tumor production [69]. The E6 of the CcarPVs contains two zinc-binding domains which show conservation with other felid PVs as well as other Carnivora species that are most closely related (Figure 6). The C-X-F-C-X_29_-C-X_2_-C motif is the conserved for the first domain; however, the second domain has one less amino acid in the lambdapapillomavirus E6 proteins compared with those of treisetapapillomaviruses C-X_2_-C-X_3_-L-X_21/23_-R-X_3_-R-X_2_C-X_2_-C. The E7 protein L-X-C/S-X-E motif which binds the pRB in the lambdapapillomaviruses has a conserved L-X-C-X-E, unlike the treisetapapillomaviruses where it is L-X-S-X-E (Figure 6). The zinc-binding domain in the E7 for members of these two genera varies in the number of residues from 34–37 nts (C-X_2_-C-X_26/28/29_-C-X_2_-C). This domain, for all of those in the E7 proteins of PVs in the *Treisetapapillomavirus* genus, and that of LwiePV1 (MH910493) [22] and CcrPV1 (HQ585856) [64] from the *Lambdapapillomavirus* genus, has a 37 residue zinc-binding motif. On the other hand, the E7 of other members of the lambdapapillomaviruses have 36 residues, except for UuPV1 (DQ180494) from snow leopard [20] which has 35 residues.

## 4. Conclusions

Through the sampling of deceased caracals in the greater Cape Town region of South Africa, we identified ten novel PV genomes from five individuals. These represent six diverse caracal PV types (CcarPV1–6) that belong to two genera, *Treisetapapillomavirus* and *Lambdapapillomavirus*. *Lambdapapillomavirus* comprises members of PVs from other felids [20,22] and the Carnivora species, whereas *Treisetapapillomavirus* previously only comprised Leptonychotes weddellii papillomavirus 2 (MG571089) [5] and Vulpes vulpes papillomavirus 1 (KF857586) [61]. Although these were identified from rectal swabs of deceased caracals, no obvious pathology typical of PV infections were noted. Not all PVs are associated with lesions or papillomas; for example, several of the human-infecting PV types in the *Betapapillomavirus* genus are symptomless [70].

Three of the five caracals were identified to have mixed infections of 2–3 CcarPV types. Additionally, four out of the five caracals harbored at least one CcarPV type whose genome is identical to that from another caracal. Given that PV transmission requires close direct contact, this may be representative of social interactions and family connections between the four caracals (CM75, CM91, CM93, and CM108). Alternatively, given the slow evolutionary rate of papillomaviruses, these types may have been circulating in this population for some time and transmitted through intermediary interaction partners that were not sampled in this study.

We were able to determine the host mitochondrial genomes for the five caracals which all share a 99.9–100% genome-wide pairwise identity with each other. Although we were unable to determine relatedness, a more extensive host genomic investigation of these caracals would help to shed some light on any family relationships. A recent study has shown that the Cape Peninsula caracal population has limited inward migration and appears to have high levels of inbreeding [58] which is supported in the lack of diversity seen in the mitochondrial genomes described here.

Sequence and phylogenetic analyses of these CcarPVs shows that, although these do share similarities to other PVs at a nucleotide and protein level, they are still diverse and distinct from other PVs. The identification of two lineages of the CcarPVs shows that there are diverse PVs circulating within an individual as well as within this caracal population. Notably, caracal CM91 from which CcarPV1, CcarPV5, and CcarPV6 were recovered harbored PV types belonging to the two lineages (*Lambdapapillomavirus* and *Treisetapapillomavirus*). This is similar to the pattern seen for the domestic cat PVs [18] as well as some other mammal PVs [1,5,71,72].

A unique non-coding region between the E2 and L2 ORFs is present in CcarPV1–4, other felid PV members, as well as CcrPV1 [64] from a spotted hyena (in the *Lambdapapillomavirus* genus), likely resulting from an expansion event that may have occurred in a shared ancestor. This region likely plays a regulatory or functional role, given the conserved nucleotide motifs present across members of this PV lineage. Four conserved motifs were identified in this region that are likely single-stranded DNA-binding domains; however, more research is needed to elucidate the biological importance of these and this insertion/expansion region. Since Rector et al. [20] demonstrated evidence of a long co-speciation history for the feline PVs, several new lineages of domestic cat PVs have been identified. It is, therefore, possible that several lineages have coevolved with their felid hosts for some time. These findings, together with the diverse caracal PVs from this study, also highlights possible host switching and/or recombination, leading to the emergence of these polyphyletic lineages.

Overall, the findings in this study expand the known felid PV diversity, demonstrate the utility of using rectal sampling for identifying PVs, as well as host mitochondrial genomes, and provide broader insights into PV dynamics in wild felid populations.

## Figures and Tables

**Table 1:** Summary of PVs (full genome and partial sequences available in GenBank) that have been identified in felids. N/A—not avaliable/unknown.

Type/Species/Genus	Source	Accession Number	Collection Date	Country	Nucleotide Completeness	Isolation Source	Reference
Acinonyx jubatus papillomavirus 1 (AjuPV1)/unclassified	Cheetah (*Acinonyx jubatus*)	MG552617-MG552632, MG552634-MG552638, MG552640-MG552642, MG552644-MG552651, MG552653-MG552668	2014/2015	Namibia	Partial	Oral lesion	[23]
Leopardus wiedii papillomavirus 1 (LwiePV1)/unclassified/*Lambdapapillomavirus*	Margay (*Leopardus wiedii*)	MH910493	2017	Costa Rica	Complete	Skin lesion	[22]
Lynx rufus papillomavirus 1 (LrPV1)/*Lambdapapillomavirus 1*/*Lambdapapillomavirus*	Bobcat (*Lynx rufus*)	AY904722	N/A	USA	Complete	Oral lesion	[20]
Panthera leo persica papillomavirus 1 (PlpPV1)/*Lambdapapillomavirus 1*/*Lambdapapillomavirus*	Asiatic lion (*Panthera leo persica*)	AY904724	N/A	USA	Complete	Oral lesion	[20]
	Cheetah	KP760482, KP760483	2014	Namibia	Partial	Oral lesion	[23]
	African lion (*Panthera leo*)	KP760481	2014	Namibia	Partial	Oral lesion	[23]
		MG552616	2014	South Africa	Partial	Oral lesion	[23]
		MG552633	2014	South Africa	Partial	Oral lesion	[23]
		MG552639	2014	Namibia	Partial	Oral lesion	[23]
		MG552652	2017	South Africa	Partial	Oral lesion	[23]
		MG552669	2017	South Africa	Partial	Oral lesion	[23]
	Asian tiger (*Panthera tigris tigris*)	MG552643	2015	South Africa	Partial	Oral lesion	Unpublished
Uncia uncia papillomavirus/unclassified	Snow leopard (*Panthera uncia*)	OR355483	N/A	USA	Partial	Skin lesion	[25]
		MT799783	2012	Mongolia	Partial	Rectal swab	[24]
Uncia uncia papillomavirus 1 (UuPV1)/*Lambdapapillomavirus 1*/*Lambdapapillomavirus*	Snow leopard (*Panthera uncia*)	DQ180494	N/A	USA	Complete	Oral lesion	[20]
Puma concolor papillomavirus 1 (PcPV1)/*Lambdapapillomavirus 1*/*Lambdapapillomavirus*	Puma (*Puma concolor*)	AY904723	N/A	USA	Complete	Oral lesion	[20]
Bos taurus papillomavirus 14 (BVP14) /*Deltapapillomavirus 4*/*Deltapapillomavirus*	Domestic cat (*Felis catus*)	KP276343	2012	USA	Complete	Skin lesion	[19]
Feline sarcoid-associated papillomavirus/unclassified	Domestic cat (*Felis catus*)	FJ977616	2008	USA	Partial	Skin lesion	[26]
Felis catus papillomavirus 1 (FcaPV 1)/*Lambdapapillomavirus 1*/*Lambdapapillomavirus*	Domestic cat (*Felis catus*)	AF480454	N/A	N/A	Complete	Skin lesion	[11]
Felis catus papillomavirus 2 (FcaPV 2)/*Dyothetapapillomavirus 1*/*Dyothetapapillomavirus*	Domestic cat (*Felis catus*)	EU796884	2007	Germany	Complete	Skin lesion	[14]
		LC612600	N/A	Japan	Complete	Skin lesion	[27]
		KP868617	2014	Italy	Partial	Skin lesion	[28]
Felis catus papillomavirus 3 (FcaPV 3)/*Taupapillomavirus 3*/*Taupapillomavirus*	Domestic cat (*Felis catus*)	JX972168	2010	New Zealand	Complete	Skin lesion	[9]
		KY825188	2012	USA	Complete	Oral lesion	[29]
		KP868618	2014	Italy	Partial	Skin lesion	[28]
		LC333418	2013	Japan	Partial	Skin lesion	[30]
		OP321266	2019	Turkey	Partial	Skin lesion	Unpublished
		HM130736	1997	USA	Partial	Skin lesion	[31]
Felis catus papillomavirus 4 (FcaPV 4)/*Taupapillomavirus 3*/*Taupapillomavirus*	Domestic cat (*Felis catus*)	KF147892	2011	New Zealand	Complete	Oral lesion	[12]
		LC333412	2013	Japan	Complete	Skin lesion	[30]
		EF447284	2007	USA	Partial	Skin lesion	[15]
		LC333413	2014	Japan	Complete	Skin lesion	[30]
		MZ357115	2020	China	Partial	Oral swab	[32]
		HM802139	2010	New Zealand	Partial	Skin lesion	[33]
Felis catus papillomavirus 5 (FcaPV 5)/unclassified Taupapillomavirus/*Taupapillomavirus*	Domestic cat (*Felis catus*)	KY853656	2016	New Zealand	Complete	Skin lesion	[10]
		LC432492, LC432493	2017	Japan	Partial	Skin lesion	[34]
Felis catus papillomavirus 6 (FcaPV 6)/unclassified *Taupapillomavirus*/*Taupapillomavirus*	Domestic cat (*Felis catus*)	MN857145	2020	Australia	Complete	Skin lesion	[13]
Felis catus papillomavirus 7 (FcaPV 7)/unclassified *Dyothetapapillomavirus*/*Dyothetapapillomavirus*	Human skin of domestic cat owner	OL310516	N/A	USA	Complete	N/A	[16]
Felis catus papillomavirus unclassified/unclassified/*Taupapillomavirus*	Domestic cat (*Felis catus*)	OQ836188	2022	New Zealand	Complete	Skin lesion	[17]
		OP762604	2022	New Zealand	Partial	Skin lesion	[35]
Felis catus papillomavirus unclassified	Domestic cat (*Felis catus*)	FJ222327	2008	New Zealand	Partial	Skin lesion	[36]
Felis catus papillomavirus unclassified	Domestic cat (*Felis catus*)	KX345934	2016	New Zealand	Partial	Skin lesion	[35]
Felis catus papillomavirus unclassified	Domestic cat (*Felis catus*)	ON017788	2022	New Zealand	Partial	Skin lesion	[35]
Felis catus papillomavirus unclassified	Domestic cat (*Felis catus*)	GU724683	2009	New Zealand	Partial	Oral lesion	[37]
Human papillomavirus 182 (HPV182)/*Betapapillomavirus 2*/*Betapapillomavirus*	Domestic cat (*Felis catus*)	GQ916646	2001	USA	Partial	N/A	[38]
Human papillomavirus 9 (HPV9)/*Betapapillomavirus 2*/*Betapapillomavirus*	Domestic cat (*Felis catus*)	EF608232	N/A	New Zealand	Partial	Skin lesion	[39]

**Table 2:** Sample information for PV-positive caracals from this study.

Animal ID	Sampling Date	Age Class	Sex	Cause of Mortality	Latitude	Longitude	Caracal Caracal Papillomavirus (CcarPV) [Accession Number]
CM93	23 April 2022	Adult	Female	Hit by car	−34.009083	18.348333	CcarPV1 [OR915585] CcarPV2 [OR915587]
CM108	18 May 2023	Kitten	Female	Disease or pesticides	−34.1066937	18.3710802	CcarPV1 [OR915586] CcarPV2 [OR915588] CcarPV3 [OR915589]
CM75	1 March 2021	Subadult	Male	Hit by car	−34.083054	18.427006	CcarPV5 [OR915591]
CM91	10 April 2022	Adult	Female	Hit by car	−34.101580	18.417405	CcarPV1 [OR915584] CcarPV5 [OR915592] CcarPV6 [OR915593]
CM111	2 June 2023	Adult	Male	Poached	−34.002037	18.445959	CcarPV4 [OR915590]

## Data Availability

The PV genome sequences were deposited under accession # OR915584-OR915593 and the caracal mitochondrial genomes under accession # PP566117- PP566121. The mapped raw reads to these PV genomes are deposited under BioProject #PRJNA1045660, BioSample # SAMN38451862-SAMN38451866 and SRA # SRR26982246-SRR26982250, and the caracal mitochondrial genomes under BioProject PRJNA1033669-PRJNA1033673, BioSample # SAMN38451862-SAMN38451866, and SRA # SRR28492289-SRR28492293.

## Supplementary Materials

The following supporting information can be downloaded at: https://www.mdpi.com/article/10.3390/v16050701/s1 Supplementary Data S1: Pairwise identity matrices for the full genome and E1, E2, E3, E6, E7, L1, and L2 protein sequences of the CcarPVs with their most closely related PV representative sequences.
